# 

**Published:** 1939

**Authors:** 


					Vol. LVI. No. 211
SPRI1
fG, 193!?.
The Bristol ^ \
Medico-Chirurgical Journal
PUBLISHED UNDER THE AUSPICES OP
THE BRISTOL MEDICO-CHIRURGICAL SOCIETY.
Editor: J. A. NIXON, C.M.G., M.D., F.R.C.P.
WITH WHOM ABE ASSOCIATED
E. W. HEY GROVES, D.Se., M.S., F.R.C.S.,
A. E. ILES, O.B.E., M.B., B.S., F.R.C.S?
A. RENDLE SHORT, M.D., B.S., B.Sc., F.R.C.S.
E. WATSON-WILLIAMS, M.C., Ch.M., F.R.C.S., Assistant EdiivrJ o,
vy Ks
Editorial Secretary: A. L. FLEMMING, M.B., Ch.B.^.A^/x, _ K ' ,
X.
BRISTOL: J. W. ARROWSMITH LTD., QUAY ST. & SMALL ST.
London : J. W. Arrowsmith (London) Ltd.
Fulwood House, Fulwood Place, High Holborn, W.C. 1
Price Three Shillings net.
Printed in Great britath,

				

